# The Physician’s Pocket, Dose, and Symptom Book

**Published:** 1861-09

**Authors:** 


ART. VII.—*The Physician's Pocket Dose, and Symptom Book; containing the Doses and Uses of all the principal articles of the Materia Medica, and Officinal Preparations; also Tables of Weights and Measures, etc., etc.* By JOSEPH H. WYTHES, A.M., M.D., author of the Microscopist, etc. Third edition, 18mo., pp. 244. Philadelphia: Lindsay and Blakiston, 1861.

WHEN a work passes through successive editions we naturally expect to find the latest edition more accurate in every respect than those which preceded it. The errors of the past, if any existed, may then be forgotten in the correctness of the present. We imagined, therefore, that the third edition of Dr. Wythes's book might possess special claim to our notice, and that we might have it in our power to commend it as a useful little work of reference. We were not prepared to find such negligence, exhibited in numerous typographical errors, which imply either indifferent proof-reading or carelessness on the part of the author. In a cursory glance at its contents we detect many inaccuracies and omissions, a few of the more glaring of which we cite by way of illustration.

In Chapter IV., "Table of Poisons and their Antidotes," no distinct mention is made of poisoning by strychnia, though its importance demands special consideration. The heading, "Opium and other Narcotics," perhaps is intended to include the subject of strychnia and kindred alkaloids. The classification of the Materia Medica is that suggested by Headland, which had its day of popularity, but is not destined to be adopted at all universally. These, however, are minor points of distinction between therapeutists, and not of so much consequence as positive errors.

And first as to officinal names, of which the author evidently makes a feature in his work, and a feature they really may be considered when printed as some of them are. For instance, we have liquor plumbi subacet*as*, aqua camphor*a*, aqua cinnamo*ni*, emplastrum ammoniac*um*, emplastr*i*um assafœtidæ, infus*is* for infusum, quin*æ* sulphas, morphi*a* acetas, tinctura gallæ aromatic*us*, tinctura valerianæ ammoniat*i*, pilulæ aloes et myrrh*a*, aqua acid*a* carbonici, calcis hydrat*us*, emplastrum picis cum cantharid*æ*. As the author states in his preface that "the list now embraces *every* officinal remedy and preparation," we have thought it worth while to look for some of the ordinary articles of the pharmacopœia, and we find several of them either missing or not printed in accordance with the nomenclature of that authority. Argenti nitras fusus is omitted entirely, so also are adeps, ferri pulvis, liquor plumbi subacetatis dilutus, potassa cum calce, potassæ carbonas impurus, liquor ferri iodidi, and a few others.

If our own national standard has formed the basis of this little work, and we find nothing in title-page or preface to tell us the contrary, from what possible source of faulty information can the author have obtained many of the items which, he considers, "render it a *vade mecum*, in which the most useful information is compressed into the smallest possible compass"? Every man who professes to the world that he follows the United States Pharmacopœia is bound to respect its authority.

We find, for instance, aether sulphuricus instead of æther; aconitina, the very poisonous active principle, given as a synonym to aconitum, a most inexcusable mistake; sulphur sublimatum instead of sulphur; spiritus ætheris *sulphurici* compositus, etc. Vinum antimonii is made of tartar emetic ℈ij, and sherry wine Oj, instead of ℈j to f℥x; and liquor potassæ

arsenitis, (Fowler's solution) 80 grains each of arsenious acid and carbonate of potassa, instead of 64 grains, to the pint of water. Emplastrum ammoniacum, (ammoniacum plaster,) and emplastrum ammoniaci, (ammoniac plaster,) are mentioned as if they were two distinct plasters. Then we have ferri iodidi solutio, instead of liquor ferri iodidi. Liquor sodæ chlorinatæ (Labarraque's solution) is wrongly made, and separated from liquor Labarraquii chloro-sodicus, which is of a very faulty nomenclature and not officinal.

Iodide of potassium is not made according to the Pharmacopœia method, by the combination of iodine and potassium, but by adding iodide of iron and carbonate of potassa. Acidum arseniosum is not mentioned as ever given in pill; and ratsbane, a common name for arsenic, is carried over to the side of strychnia as its synonym. Under quinia, we are told "see cinchona," but we look there in vain for a word about quinia; so also under sassafras, "see decoction," but there is no such heading as "decoction" in any part of the book.

Hydrargyrum ammoniatum, which is in reality an amidide of calomel, ($Hg\ Cl, NH_2$) is spoken of as "a binoxide, combined with bichloride of mercury and ammonia, forming a triple salt." Extractum sarzae occurs as well as decoctum sarzae compositum; and we find under the letter S, sarsaparilla instead of sarza. We had hoped that the old term "deobstruent" had been allowed to die a natural death long since, but we here find it revived; we confess we have no very definite idea what it means as used by the author. "Infusum cinchonæ cum succo limonum" is a wholly useless addition to the list, unless it has, we may suggest, something added to it, such as "et saccharo albo," by way of giving it lengthiness and pleasantness.

The author errs in stating (p. 103) that Magendie's solution of *sulphate* of morphia contained 16 grains of that salt to a *fluidrachm* of water; he employed the *acetate*, not the sulphate, and in the strength of 16 grains to the *fluidounce*—a point of material importance, so far as the dose is concerned.

The faults of the index, of course, correspond with those of the body of the work. We have purposely omitted allusion to the chapter on symptomatology, general pathology, etc., because we deemed the other portions more important. We must protest, however, against "dulness of sound," "wooden sound," etc., as being the "effects of percussion," (p. 196;) or "weak respiration" or "bronchial respiration," as the "effects of auscultation." In conclusion, we may suggest that the author should revise the posological parts of the volume. The doses are generally accurate, but occasionally medicines are employed for therapeutical actions not mentioned by the author, and therefore with slightly different doses.

It was not our intention to notice Dr. Wythes's book so fully; we have pointed out errors in it in a kindly spirit, because we have thought its usefulness to the profession might be augmented by revision for a succeeding edition. There is a convenience frequently in having a little companion in the pocket for reference, when one's memory is at a loss for names or doses.

